# Structural Insights into the Osteopontin-Aptamer Complex by Molecular Dynamics Simulations

**DOI:** 10.3389/fchem.2018.00002

**Published:** 2018-01-30

**Authors:** Giovanni La Penna, Riccardo Chelli

**Affiliations:** ^1^Istituto di Chimica dei Composti Organometallici, Consiglio Nazionale delle Ricerche (CNR), Florence, Italy; ^2^Dipartimento di Chimica, Università di Firenze, Florence, Italy

**Keywords:** osteopontin, aptamers, intrinsically disordered proteins, molecular models, ion condensation

## Abstract

Osteopontin is an intrinsically disordered protein involved in tissue remodeling. As a biomarker for pathological hypertrophy and fibrosis, the protein is targeted by an RNA aptamer. In this work, we model the interactions between osteopontin and its aptamer, including mono- (Na^+^) and divalent (Mg^2+^) cations. The molecular dynamics simulations suggest that the presence of divalent cations forces the N-terminus of osteopontin to bind the shell of divalent cations adsorbed over the surface of its RNA aptamer, the latter exposing a high negative charge density. The osteopontin plasticity as a function of the local concentration of Mg is discussed in the frame of the proposed strategies for osteopontin targeting as biomarker and in theranostic.

## 1. Introduction

Osteopontin (OPN) belongs to the class of matricellular proteins involved in tissue remodeling (Frangogiannis, 2012; Kahles et al., 2014). After injuries in tissues, cell replication is induced by many extracellular factors that become over-expressed. OPN expression increases when cells replication is induced and when collagen extracellular matrix has to be reconstructed to stabilize the cells in the new tissue. Knock-out of OPN gene (SPP1 in human) eliminates the reconstruction of collagen. An increase of OPN concentration in serum is a clear indication of myocardial remodeling, both after myocardial infarction and in case of pathological cardiac hypertrophy and fibrosis.

OPN is the target of several therapies that have been proposed to interfere with the signaling process activating or modulating cell-cell or cell-matrix interactions. A proposed theranostic agent is based on the specific interactions between osteopontin and an RNA aptamer (Mi et al., 2009) (OPN-R3, hereafter). These RNA aptamers can be easily synthesized *in vitro* and supported over gold nanoparticles (Liu and Lu, 2006) containing fluorescent probes or dyes. The nanoparticles can therefore be used to monitor the concentration of OPN in serum or to deliver drugs in the extracellular region where the OPN concentration is pathologically high.

OPN belongs to the class of intrinsically disordered proteins (IDPs). The protein of 300 aminoacids has no three-dimensional structure in water (Kurzbach et al., 2013; Platzer et al., 2015). The many interactions between OPN and other proteins and cofactors indicate that the lack of structure enables OPN to perform different functions (Sodek et al., 2000; Mazzali et al., 2002). Among these interactions we mention: (i) integrin receptors with the RGD domain (Christensen et al., 2010); (ii) CD44 domain and collagen, with highly negative polysaccharides involved (Ponta et al., 2003); (iii) post-translational modifications activating different biochemical pathways (Weber et al., 2002), including biomineralization (Li and Wang, 2012); (iv) interactions with cations (Ca, Mg) modulating OPN activity (Hu et al., 1995); (v) OPN antibodies inhibiting the rheumatoid arthritis propagation (Du et al., 2008) (PDB 3CXD). We shall indicate this framework of diverse interactions as OPN plasticity (Peysselon et al., 2011).

The role of IDPs in biomineralization has been reported (Kalmar et al., 2012) and OPN shares this property with many IDPs (Hunter et al., 2010). The interaction with Ca^2+^ cations has been hypothesized in the N-terminus of OPN, characterized by a significant density of D/E residues (single-letter aminoacid coding is mostly used, hereafter). The protein contains, in the post-processed 17–300 chain, 47 D, 24 E, 18 K, and 9 R, for a total charge at physiological pH of −44. The high polarization of charge in OPN (the charge of first 100 residues is −26) indicates that the interactions of OPN with macromolecular partners is associated to a collective rearrangement of the N-terminus consequent to the approach of the target molecule by OPN.

**Figure 1:**
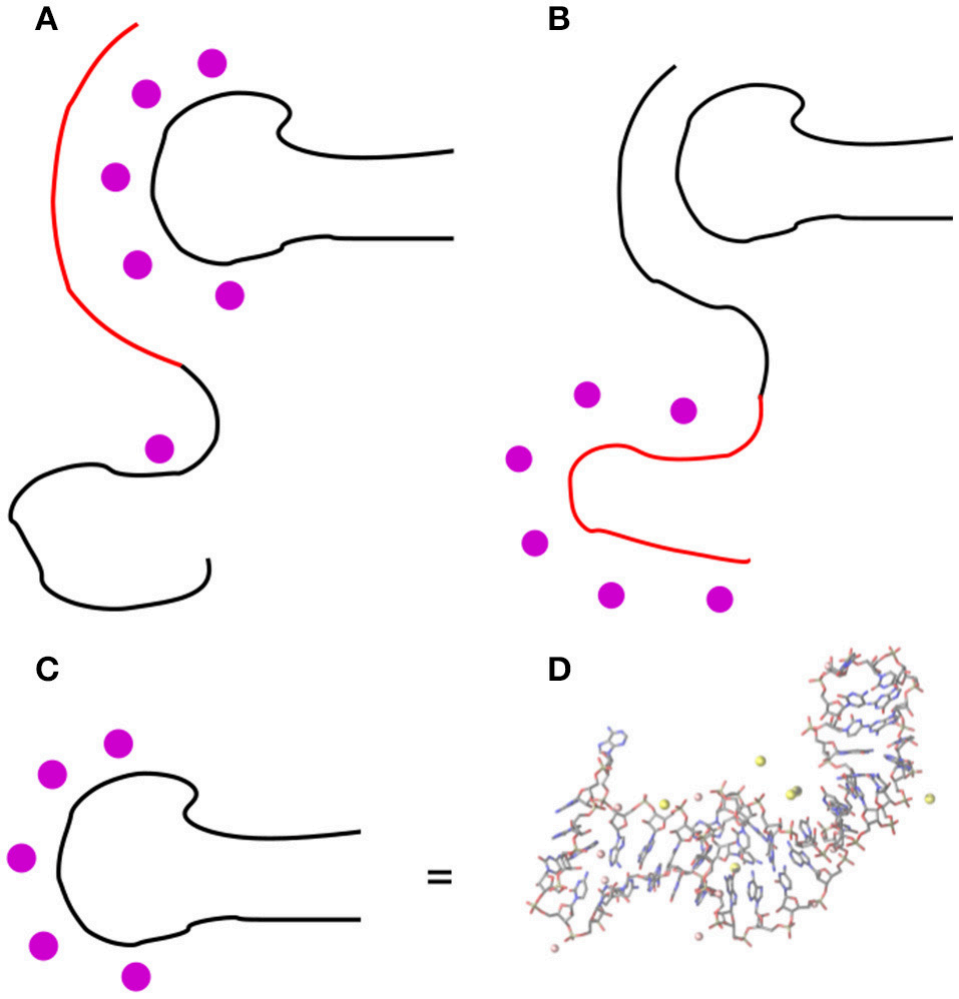
Two possible arrangements of the OPN coil in the OPN-(OPN-R3) complex: **(A)** the negatively charged N-terminus (in red) forms an extended array of electrostatic interactions (like salt bridges) with the RNA phosphate groups, mediated by mono- and divalent cations (magenta circles); **(B)** the negatively charged N-terminus is repelled by RNA and the C-terminus is forced for non-electrostatic interactions with RNA. In **(D)** the RNA drawing of OPN-R3 **(C)** is expanded into the atomic structure obtained as the final configuration of OPN-R3 simulated for 23.4 ns with MgCl_2_ (see also Figure S4). RNA is represented as sticks; C is gray, N blue, O red; H atoms are not displayed; Mg and Na ions within 0.5 nm from OP are represented as pink and yellow spheres, respectively. Bond and atomic radii are arbitrary. The stem-loop RNA region (11–24) is on the left-hand side and the RNA termini (1–2 and 34–40) are on the top-right. The VMD program (Humphrey et al., 1996) is used for all the molecular drawings.

As for the binding of OPN to RNA, two possible arrangements of the approaching OPN random coil can be hypothesized, as drawn in Figure 1.

In the first hypothesis, the negatively charged N-terminus of OPN forms an extended array of electrostatic interactions with mono- and divalent cations more or less adsorbed on the RNA molecule (the phosphate groups). In the second hypothesis, the negatively charged N-terminus is repelled by RNA and the interactions between OPN and RNA are non-electrostatic, even though they can still involve the cations adsorbed on the RNA molecule.

The interactions between OPN and heparin, the latter a polysaccharide with high negative charge density, have been recently studied by NMR and EPR techniques (Kurzbach et al., 2014, 2017). These data are consistent with the formation of a patch of complementary charges, with an unfolding-upon-binding mechanism that releases the compaction of OPN in the absence of any partner. This kind of assembly process resembles that drawn in Figure 1B. On the other hand, all NMR and EPR studies performed so far are in the absence of divalent cations.

In this work, we aim at providing models to understand the OPN plasticity, with the specific goal of understanding how OPN interacts with its aptamer OPN-R3 (Mi et al., 2009), in the presence of naturally occurring divalent cations. Therefore, still in the frame of the electrostatic hypothesis that is generally assumed for IDPs, we exploit the other possible mechanism (Figure 1A), on the basis of the expected strong effect of divalent cations in the RNA function.

Despite the limited time-length (55 ns) of molecular dynamics simulations performed for the large macromolecular complex (more than 500,000 atoms, including the model of ionic atmosphere), the structural models reported here provide a possible alternative frame compared to that observed with no divalent cations, and assist to a better prediction of the interactions of OPN when it is bound to the aptamer and to the theranostic nanoparticle that supports the aptamer.

## 2. Methods

We built 6 initial configurations for the OPN-(OPN-R3) complex with a low number of intermolecular contacts (see Figure S1). Explicit water molecules and counterions were included. All the 6 initial structures were simulated with molecular dynamics (MD) in the NPT statistical ensemble at *T* = 300 K and *P* = 0 bar, in a range of physiological salt concentrations conditions, for 10 ns. Two of these configurations were simulated for 55 ns. The system size is summarized in Table 1. The size, in terms of number of atoms, is significant because of the requirement of modeling the ionic atmosphere including divalent cations and because of the size of the initial models for the disordered OPN protein. This size prevents the collection of adequately long MD simulations (likely 1 μs) and of more independent or statistically combined trajectories (likely more than one hundred). Therefore, we concentrate the modeling to discriminate between the two different complex topologies described in Figure 1.

**Table 1:** Number of atoms in the simulated systems.

**Trajectory**	**Species**	**Part 1**	**Part 2**	**Part 3**
OPN-R3	Na	243	–	243
	Mg	20	–	0
	Cl	244	–	204
	wat	108,645	–	108,705
	total	327,842	–	327,842
**1**	Na	366	384	406
	Mg	20	11	0
	wat	172,307	172,298	172,287
	total	523,319	523,247	523,159
**2**	Na	366	368	406
	Mg	20	19	0
	wat	172,279	172,278	172,287
	total	523,235	523,227	523,159
**3**	Na	366	374	406
	Mg	20	16	0
	wat	172,281	172,277	172,261
	total	523,241	523,209	523,081
**4**	Na	366	376	406
	Mg	20	15	0
	wat	172,314	172,309	172,294
	total	523,340	523,300	523,180
**5**	Na	366	386	406
	Mg	20	10	0
	wat	172,250	172,240	172,230
	total	523,148	523,068	522,988
**6**	Na	366	368	406
	Mg	20	19	0
	wat	172,294	172,293	165,893
	total	523,280	523,272	503,977

*Atoms in OPN protein are 4,289 (284 aminoacids) and in OPN-R3 aptamer are 1,280 (39 bases). The number of chloride ions is always 323 for OPN-(OPN-R3) complex. The amount of water solvent (wat) is in molecules*.

All the details of the construction and simulations are reported in the Supplementary Material. Here, we summarize only the most important points. The AMBER PARM99 force field (Want et al., 2000) was used in all simulations performed with the LAMMPS code (Plimpton, 1995). As for cations, we used a recent force field for divalent cations (Duarte et al., 2014), in addition to the usual parametrization of NaCl (Aaqvist, 1990). This force field reduces the charge density over the divalent cation, thus avoiding the usual problem of negatively charged (carboxylate and phosphate) groups collapsing over the highly-charged points. For long-range electrostatic interactions, we used the damped and shifted force method (Fennell and Gezelter, 2006) implemented in LAMMPS. For a critical review about this method compared to more accurate, but computationally slower, methods (see Cisneros et al., 2014).

After the construction of the OPN-R3 structure, the structure of the ionic layers around it has been simulated by MD in explicit water for about 23 ns. The analysis confirmed most of the observations reported in the literature about the structure of mono- and divalent cations around RNA stem-loop configurations. This analysis is reported in the Supplementary Material.

As for the construction of initial configurations for the OPN-(OPN-R3) complex, the configuration of RNA with Mg simulated after 18 ns was used. This configuration, as shown by the analysis described in the Supplementary Material, has 9 Mg cations adsorbed. These cations were kept for the construction of the initial complex samples.

A random configuration for the OPN protein was built and put in contact with the OPN-R3 selected structure. A set of non overlapping OPN-(OPN-R3) configurations was generated by using random temperature Monte Carlo trajectories (La Penna, 2003; La Penna et al., 2004) (MC-RW), summarized in the Supplementary Material. The OPN C-terminus (N284) and the RNA OPN-R3 aptamer were mutually oriented and kept fixed in space in 6 samples, differing for the initial position of the C-terminus in front of the center of the RNA stem. The entire protein chain of OPN randomly moved around the RNA molecule. For each sample, we choose the first configuration displaying a manifest contact between OPN and OPN-R3 as the starting configuration for the following MD simulation (Figure S1, Supplementary Material). Therefore, once a configuration mimicking a possible initial contact between OPN and OPN-R3 was selected within the 6 MC-RW performed, MD simulations of the OPN-(OPN-R3) complex were performed in explicit water and with explicit counterions.

The chosen Mg concentrations (see Table 2) are consistent with the range of concentrations for divalent cations (Mg^2+^ and Ca^2+^) in the simulated body fluid (Oyane et al., 2003) used for *in vitro* OPN-RNA binding (Mi et al., 2009) and biomineralization studies (Kalmar et al., 2012). After performing 10 ns of simulation in the NPT statistical ensemble for the 6 samples, each at the 3 different salt concentrations, we performed longer simulations (55 ns) for two selected samples and ionic environments.

**Table 2:** Equilibrium concentrations (mM) of Na and Mg, and simulation times in the different samples.

	**Part 1**			**Part 2**			**Part 3**		
OPN-R3	121	10	(10) 13.4	–	–	–	121	0	(5) 16.2
**1**	114	6	(5) 6.1	120	3	(5) 5.2	127	0	(5) 5.0
**2**	114	6	(5) 5.0	115	6	(5) 5.8	127	0	(5) 6.0
**3**	114	6	(5) 8.2	117	5	(5) 7.5	127	0	(5) 5.2
**4**	114	6	(5) 5.7	118	5	(5) 5.5	127	0	(5) 5.0
**5**	114	6	(5) 5.0	121	3	(5) 5.4	127	0	(5) 5.2
**6**	114	6	(5) 5.5	115	6	(5) 5.7	127	0	(5) 6.0

*Equilibration times (within brackets) and sampling times are in ns. Volume fluctuations in the last 5 ns of all simulations are within 0.1%*.

## 3. Results and discussion

### 3.1. OPN-R3 aptamer

The settling of the ionic atmosphere around the stem-loop structure of OPN-R3 (see Supplementary Material), showed that a high degree of screening of RNA is performed by both Na condensation (within 3 nm) and Mg adsorption over the RNA surface (within 0.28 nm). The analysis shows that Mg replaces Na in the condensation layer, but the Mg ions are more structured around the phosphate groups of RNA.

The analysis of the OPN-R3 aptamer simulations, shows that, as expected, the simulation time is not long enough to sample all the structural changes occurring in the RNA fragment during the stabilization of the layer of cations (see discussion of the root-mean square deviation and solvent accessible surface area). We remark that the aim of the model is to probe the general effect of the formed layer on the protein-RNA interactions, regardless of specific interactions that can be adequately sampled with atomistic models of this size only using hundreds of replica in the μs time-scale.

Summarizing, the RNA molecule adsorbs 8 ± 2 divalent cations and 2 ± 1 Na cations, on average. The comparison of radial distribution function for OP-M pairs (OP being the non-phosphodiester O atom of RNA phosphate groups) with that for all pairs X-M shows that this number is dominated by OP contacts when M is Mg, but there is a significant difference when M = Na. Even when Mg is absent, the number of X-adsorbed Na is 6 ± 2, while that for OP-adsorbed Na is 3 ± 2. This difference means that Na ions are not really adsorbed, while there is exchange between the first shell and farther shells, allowing short-range interactions with groups different from the phosphate groups. This observation is consistent with a loose structure of short-range Na shells. On the other hand, in case of Mg, the number of adsorbed ions measures the extent of ion adsorption by the phosphate groups as an additional information to the ion condensation, the latter occurring within the larger distance of 3 nm (see Supplementary Material).

As a consequence of the ionic behavior summarized above (see Supplementary Material for a more detailed discussion), most of the screening contribution due to Mg is made by phosphate-adsorbed Mg (8 cations over 9, see Supplementary Material). This adsorption is a very slow process, as already noticed in 100-ns long simulations (Casalino et al., 2017). Na is more diffuse within the distance of about 3 nm, and the phosphate-adsorbed Na ions provide a small contribution to the screening positive charge (2 over 22 Na ions within 3 nm). The structure of these layers will serve as a reference for the comparison with the OPN-aptamer complex described in the following.

### 3.2. (OPN-R3)-OPN complex

As for (OPN-R3)-OPN complex, the time-evolution of different types of contacts and radial distribution functions have been analyzed. The time-length of 10 ns sampled for 6 models, allows the settling of the ionic atmosphere around each complex. On a longer time-scale (55 ns), sampled for 2 of the models, an initial stage of wrapping of the protein around the RNA fragment is clearly observed (see below). The simulation of a complete wrapping process, as well as of the possible equilibrium between wrapped and unwrapped configurations, is beyond the possibilities of these atomistic models. Therefore, we focus only on discriminating among the two mechanisms described, respectively, in Figures 1A,B.

#### 3.2.1. OPN-aptamer contacts

We first measured the number of OPN-RNA non specific contacts using a set-up already used to evaluate crystal and solution structures of several protein-RNA complexes. Then, the number of ions M (with M = Na, Mg and Cl) is used to eventually correlate time evolution of non specific contacts with the adsorption/release of counterions from the short-range layer discussed for RNA in the absence of the OPN protein partner. Similarly to the analysis of RNA, we also analyze the location of adsorbed cations, selecting O atoms in negatively charged groups of OPN protein (Oδ of D and Oϵ of E). These O atoms will be indicated as OD and OE, respectively, hereafter.

As for specific O-M contacts, the *d*_0_ value (see Equation 1 in Supplementary Material) is the same used for RNA (0.28 nm), because the position of the first peak in the radial distribution function of OP-M distance does not change when OPN is added. The same effect holds for O of carboxylate groups in OPN. As for the first evaluation (non specific contacts), the work reported in (Lejeune et al., 2005) was used as a reference. In that work, the *d*_0_ distance was 0.5 nm, involving any protein and RNA atom pairs, and a deep analysis was performed for 49 structure entries in the protein data-bank (PDB). We measured *CN* with *d*_0_ = 0.5 nm and for pairs involving non-hydrogen atoms of protein and RNA, respectively, for the 41 complexes, among the 49 PDB entries, for which it is possible to reconstruct the entire unit cell. This construction was performed with the VESTA program (Momma and Izumi, 2011). Among the 41 structures a further selection must be made to compare the number of contacts with the OPN-aptamer 1:1 complex investigated in this work. We selected structures where the ratio *R* = *n*_*aa*_/*n*_*nt*_ between the number of aminoacids in protein (*n*_*aa*_) and the number of nucleotides in the RNA partner (*n*_*nt*_) is in the range of the ratio that holds for the OPN-aptamer 1:1 complex, *R* = 284/40≃7. Therefore, we selected the 26 structures with ratio between 3 and 15. The *CN* value obtained for these 26 structures is then normalized to *CN*_*n*_ = *CN*/(*n*_*aa*_ + *n*_*nt*_), i.e., is divided by the total number of residues *n*_*aa*_ + *n*_*nt*_ in the unit cell. The periodic boundary conditions of the unit cell were used to compute the distances in the evaluation of *CN* in Equation 1 (Supplementary Material). The minimal value for *CN*_*n*_ in this set of structures is 0.002 and the maximal is 0.07. Among this data-set, the unique structure of a protein-aptamer complex is the structure 1OOA, with *CN*_*n*_ = 0.02. Hereafter, we shall use this value as the *CN*_*n*_ minimal value for the protein-RNA binding.

**Figure 2:**
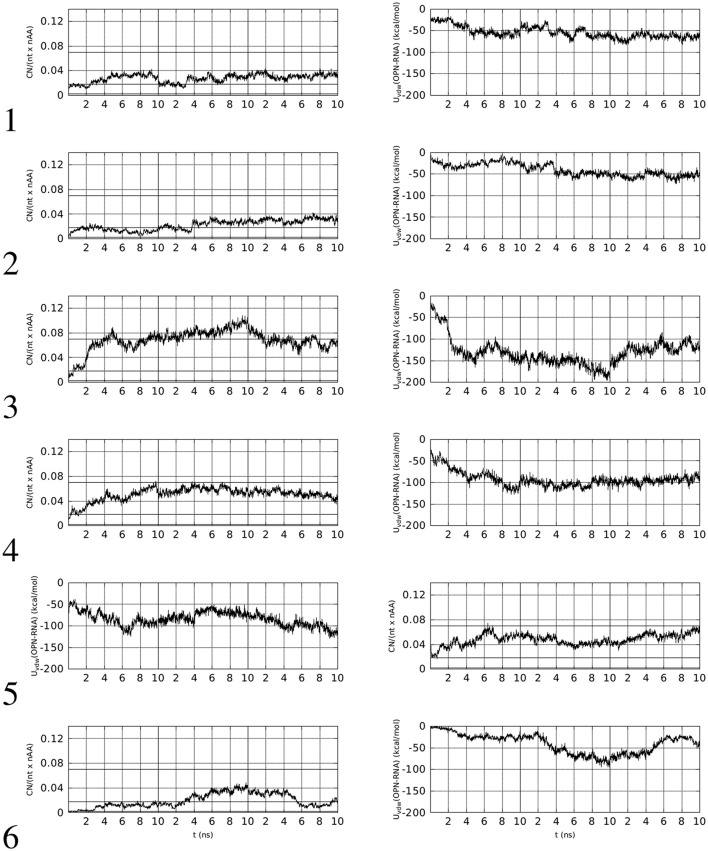
**Left:** time evolution, within the first 10 ns of simulation, of the number of contacts (*CN*_*n*_, see text for details) involving OPN and OPN-R3 non-hydrogen atoms (*d*_0_ = 0.5 nm). **Right:** time evolution of Lennard-Jones contributions to OPN-(OPN-R3) energy (*U*_*vdw*_). The vertical lines separate simulations with decreasing (from left to right) concentration of Mg. The horizontal lines in left panels indicate the range of values obtained with the identified sub-set of protein-RNA complexes in PDB, with the line at 0.02 being related to PDB record 1OOA (protein-aptamer complex).

**Figure 3:**
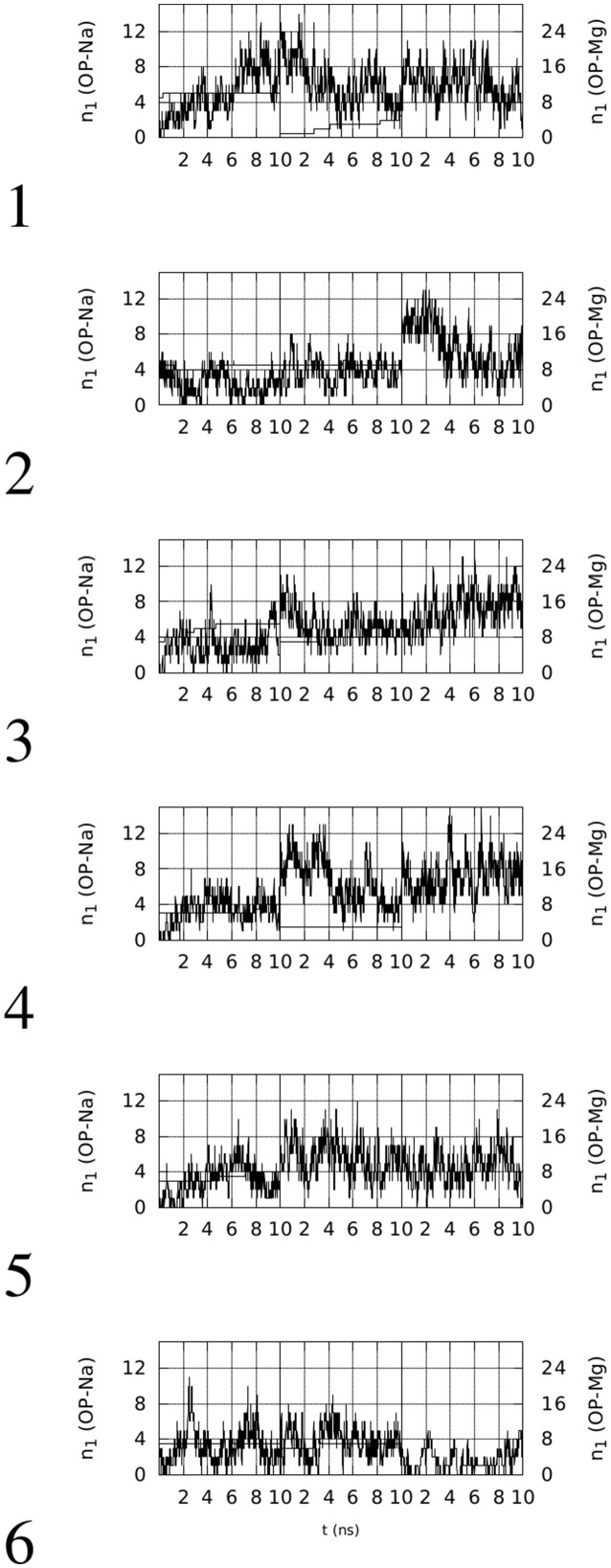
Time evolution, within the first 10 ns of simulation, of the number of contacts (*n*_1_) involving OP(RNA) atoms and Na (thin line, left *y*-axis) or Mg (thick line, right *y*-axis) ions (*d*_0_ = 0.28 nm). The vertical lines separate simulations with decreasing (from left to right) concentration of Mg.

In left panels of Figure 2, the time evolution of *CN*_*n*_ in the six simulations of OPN-aptamer complex is displayed, while the time evolution of specific O-M contacts (*n*_1_, see Supplementary Material) is displayed in Figure 3.

Left panels of Figure 2 display two features concerning the wrapping of OPN around the RNA molecule. The first feature is that in all cases there is a rapid increase in the number of contacts during the first half (5 ns) of the trajectory. This increase, with the exception of trajectory **5**, starts from values lower than the threshold displayed by the aptamer complex reported in the PDB (1OOA, 0.02), and ends within the first part of simulations at values higher than the threshold, with the exception of trajectories **2** and **6**. Therefore, 2 trajectories over 6 display, in the first part of simulation (Mg ~ 10% of Na), final values of *CN*_*n*_ higher than that of the crystallized 1OOA aptamer-protein complex. All trajectories display *CN*_*n*_ higher than the minimal value 0.002 (PDB 1B23). Concerning the second part (lower Mg concentration) of simulation, all of the trajectories end with *CN*_*n*_ higher than the 1OOA threshold.

The second feature is more specific for each trajectory. Trajectory **1** reaches a higher value at high Mg concentration (first 10 ns), then, after a sudden decrease due to the removal of phosphate-adsorbed Mg cations, the complex recovers high values of *CN*_*n*_ when other Mg cations are adsorbed by RNA (see the corresponding plot in Figure 3). The complete removal of Mg cations (last 10 ns) does not allow the unwrapping of OPN away from RNA. In trajectories **2** and **6**, the slow sticking effect of phosphate adsorbed Mg ions is also displayed, with a final partial unwrapping of OPN away from RNA when all Mg ions are removed in trajectory **6** (last 10 ns). These two trajectories display a OPN wrapping that becomes less tight, compared to the other trajectories, when no Mg is present. In complexes **3**, **4**, and **5**, higher *CN*_*n*_ values are reached in the first stage of high Mg concentration and Mg removal has no apparent effect on the OPN wrapping. In trajectory **3** the partial recovery of phosphate-adsorbed Mg occurs in the second simulation stage (central 10-ns window) and the complete removal is again not sufficient to trigger the OPN unwrapping away from RNA, even though a partial unwrapping is displayed (last 10 ns).

The behavior described above is also displayed by the non-electrostatic and non-solvation energy contribution to the formation of the OPN-RNA complex, i.e., the Lennard-Jones complexation energy. This is the sum of Lennard-Jones pair contributions to energy, with the sum extended to *i* − *j* pairs, *i* running over OPN atoms and *j* running over RNA atoms. The time evolution of this contribution is displayed in the right panels of Figure 2. The initial values, close to zero, rapidly decrease to values lower than −50 kcal/mol in all cases except trajectories **2** and **6**, that are those not reaching the threshold for *CN*_*n*_. According to this observation, the value of −50 kcal/mol for the Lennard-Jones complexation energy appears as a threshold for an irreversible wrapping. The correlation between the behavior of *CN*_*n*_ in the absence of Mg cations and that of the Lennard-Jones complexation energy is displayed by trajectories **3**, **4**, and **5**, i.e., those trajectories where the OPN wrapping is more evident and Mg cations are adsorbed by both OPN-R3 and OPN. In trajectories **3** and **4** there is partial unwrapping when no Mg is present, while in trajectory **5** the wrapping is recovered and the energy becomes lower than −100 kcal/mol.

The O-Mg contacts account for all the OPN and OPN-R3 contacts with Mg in the second half of trajectories. Differences between X (with X any atom in the solute) and O = OP, OD, OE (see Methods and Supplementary Material) as Mg adsorber, are displayed only in the first half of the trajectories, while in the second half there is no difference between the calculation including the addressed O (OP, OD, and OE) atoms and all the atoms in the OPN protein and RNA aptamer, respectively. This behavior shows that the OPN protein arranges its conformation to adapt to the constraint of possible Mg-OP and Mg-OD/OE salt bridges. A detailed analysis of the salt bridges and charge neutralization for the 6 OPN-RNA complexes simulated for 10 ns is reported in Table 3.

**Table 3:** Number of ions (Na and Mg) within first-shell layer (*d*_0_ < 0.28 nm) from different sets of atoms: all atoms in OPN protein (OPN); all O atoms in carboxylate side-chains of D and E residues in OPN [O(D/E)]; all atoms in OPN-R3 aptamer (RNA); all non phosphodiester O atoms in phosphate groups of OPN-R3 (OP); complex indicates the OPN-(OPN-R3) complex.

	**OPN**	**O(D/E)**	**RNA**	**OP**	**OPN+RNA-complex**	***Q*_*a*_(OPN)**	***Q*_*a*_(RNA)**
**1**							
Na	8(2)	5(2)	7(2)	4(2)	1(1)	−29.5	−17.5
Mg	6(0)	6(0)	10(0)	10(0)	5(0)		
Na	9(3)	5(2)	5(2)	3(2)	0(0)	−27.0	−28.0
Mg	4(0)	3(0)	3(1)	3(0)	0(0)		
Na	9(2)	6(2)	6(2)	3(2)	0(0)	−35.0	−33.0
**2**							
Na	9(3)	5(2)	2(1)	1(1)	0(0)	−28.0	−20.0
Mg	4(0)	4(0)	9(0)	9(0)	1(0)		
Na	9(2)	5(2)	4(1)	1(1)	0(0)	−25.0	−17.0
Mg	5(0)	5(0)	9(0)	9(0)	0(0)		
Na	12(3)	8(3)	5(2)	2(2)	0(0)	−32.0	−37.0
**3**							
Na	10(3)	6(2)	3(2)	2(1)	0(1)	−29.0	−19.0
Mg	5(1)	3(1)	11(0)	10(0)	5(1)		
Na	10(3)	7(3)	5(1)	3(1)	1(0)	−29.5	−19.5
Mg	3(0)	3(0)	8(0)	8(0)	1(0)		
Na	11(3)	7(2)	8(2)	4(2)	1(1)	−33.0	−31.5
**4**							
Na	15(3)	11(3)	4(1)	2(1)	1(1)	−19.5	−25.5
Mg	7(0)	7(0)	7(1)	7(1)	4(0)		
Na	13(3)	9(3)	5(2)	3(2)	0(0)	−25.0	−28.0
Mg	3(0)	3(0)	3(0)	3(0)	0(0)		
Na	14(3)	10(3)	7(2)	5(2)	1(1)	−30.5	−32.5
**5**							
Na	9(2)	6(2)	4(2)	3(1)	0(1)	−23.0	−21.0
Mg	7(1)	6(1)	8(0)	8(0)	2(0)		
Na	11(3)	7(2)	5(2)	3(1)	0(0)	−25.0	−34.0
Mg	4(0)	4(0)	0(0)	0(0)	0(0)		
Na	16(3)	12(3)	5(2)	3(2)	0(0)	−28.0	−34.0
**6**							
Na	10(3)	7(2)	3(2)	2(1)	7(3)	−30.5	−26.5
Mg	4(1)	4(1)	7(0)	7(0)	1(0)		
Na	11(2)	8(2)	3(1)	1(1)	1(1)	−17.5	−22.5
Mg	8(0)	8(0)	7(0)	7(0)	0(0)		
Na	15(4)	1(1)	2(1)	0(0)	0(0)	−29.0	−37.0

*Root-mean square errors are within parentheses. Averages are over the last 5 ns of each 10-ns long trajectory*.

The different rows for each trajectory display the number of contacts *n*_1_ (see Supplementary Material for its definition), by decreasing Mg concentration (from top to bottom). The charge of OPN and RNA reported in the last two columns is computed by assigning one half of the ionic charge to OPN and RNA, respectively, when the ion is involved in a salt bridge of type OPN-M-RNA (the fifth column). It must be noticed that the negative charge of both partners in the complex, OPN and OPN-R3 aptamer, is minimal in the presence of 10% of Mg. A significant decrease of charge (the charge becomes more negative) occurs when Mg cations are removed from the system. The exchange of divalent cations between binding sites and the bulk is very slow in RNA, as it has not been observed in 200-ns long MD simulations (Casalino et al., 2017). However, it can be observed that the initial approach between the two partners occurring when Mg is present, allows the replacement of missing Mg cations with Na cations, both in OPN and RNA. This process is similar to what has been observed for RNA (see Figure S7 and discussion in the Supplementary Material).

Summarizing, in all of the simulated trajectories 10-ns long and in the presence of about 10% of Mg cations among the positive counterions, the cations trigger the collapse of OPN onto the RNA aptamer, as shown by the initial time evolution of *CN*_*n*_ (Figure 2). Then, unwrapping becomes difficult: unwrapping occurs only in one case (**6**) over six simulations during the following 2 simulations with decreasing number of Mg cations. In this case we notice that no efficient replacement of Mg by Na occurs in the adsorbed layer.

#### 3.2.2. Extending simulations to 50 ns

**Figure 4:**
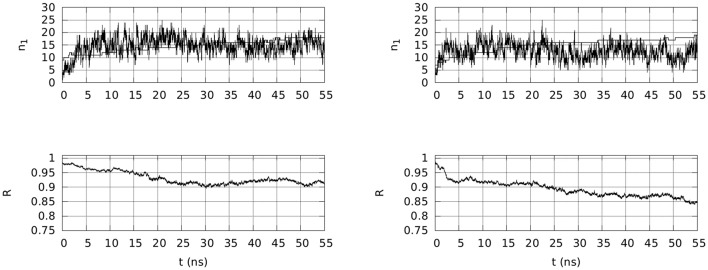
**Top:** Time evolution, within 55 ns of simulation, of the number of contacts (*n*_1_) involving any atom in OPN and OPN-R3, and either Na (thin line) and Mg (thick line) ions (*d*_0_ = 0.28 nm). **Bottom**: Time evolution, in the same time-window, of R (Equation 2 in the Supplementary Material), describing the extent of assembly between OPN and its RNA aptamer. Left panels are for trajectory **1** and right panels for trajectory **3**.

Two selected simulations, **1** and **3**, have been extended to 55 ns in the presence of Na and Mg counterions in the conditions of the simulated body fluid. In both simulations, the wrapping of OPN around OPN-R3 increases. The extent of wrapping is measured via the R quantity in Equation 2 in the Supplementary Material. In Figure 4, bottom panels, the time evolution of R shows that the wrapping is extended in both cases, but for trajectory **3** it is more extended than for **1**.

This observation clearly shows that when the initial (the first 10 ns) wrapping is moderate (see Figure 2, trajectory **1**), the wrapping is moderate also over a longer time-scale (R~0.9). On the other hand, when the initial wrapping is large (trajectory **3** in Figure 2), the two partners continue the slow build-up of the wrapped assembly.

In Figure 4, top panels, the time evolution of the number of adsorbed cations (*n*_1_, Na in thin line, Mg in thick line) is displayed. Even if in both cases the number of Mg cations that is adsorbed is almost the total number of Mg ions in the sample (18 over 20 in both cases), the time evolution is markedly different in the two cases. In the first case (**1**) the Mg adsorption is not yet completed at 55 ns and consequently the number of adsorbed Na ions is still kept at the initial level (*n*_1_ = 15–16). In the second trajectory (**3**) the number of adsorbed Na ions is significantly decreased when the number of adsorbed Mg ions is stable at 18. This exchange between Mg and Na in the adsorbed layer (*d*_0_ < 0.28 nm) occurs when the wrapping of OPN around OPN-R3 displays values of R lower than 0.9.

The time evolution of solvent accessible surface area (SASA) for OPN and OPN-R3 is also different in the two cases (data not shown). The SASA of RNA within the first 20 ns of simulation is very similar in the two cases, but during the last 20 ns the SASA(OPN-R3) for **3** is significantly larger than for **1**, thus indicating a partial RNA unfolding upon the larger wrapping of **3**. The time evolution of RMSD (Figure S8) displays, for both 55-ns trajectories, a similar behavior. The RNA fragment is kept within the same extent of deviation obtained after the first 10 ns. In trajectory **1**, the fragment is even slightly restructured at the end of the trajectory, compared to trajectory **3**. The OPN N-terminus becomes rigid after about 20 ns in both trajectories, and the C-terminus appears as following the collapse of the N-terminus, with a time delay that is larger for **3** than for **1**. In both trajectories, the C-terminus becomes more rigid after 40 ns.

Following the analysis performed for the OPN-(OPN-R3) complex for the first 10 ns of simulation (Table 3), it is possible to measure the effective charge of the OPN-RNA complex accounting for the adsorption of Mg and Na cations within the first peak of the radial distribution function, that is within the distance *d*_0_ = 0.28 nm. For trajectory **1**, 18 Mg ions are adsorbed among the total amount of 20, while only 16 Na ions among the total amount of 366 are adsorbed. In case of trajectory **3**, the number of adsorbed Mg ions is the same (see above), but that of Na ions is significantly smaller (11) when averaged over the last ns. Nevertheless, the final values of *n*_1_ are similar, 16 and 15 for Na in, respectively, **1** and **3**.

The calculation of *Q*_*eff*_ (see Supplementary Material) gives −17.5 and −13.5 for OPN and OPN-R3, respectively, in case **1**, and −16.5 and −27.5 for **3**, respectively. In both cases the negative charge of the assembly is strongly screened at short distances: 37 and 53%, respectively, of the total bare charge of −44−39 = −83.

**Figure 5:**
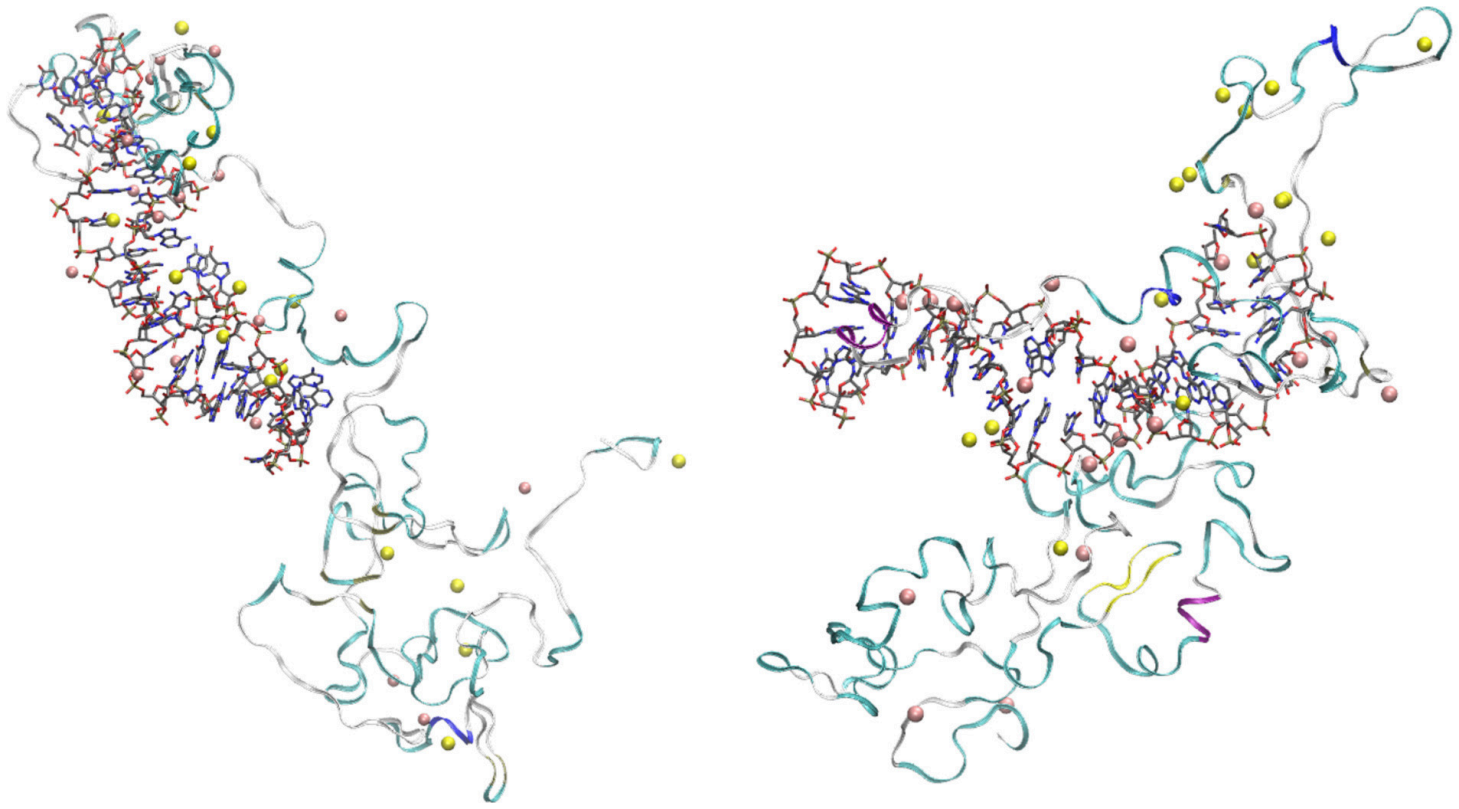
Final configurations of OPN-(OPN-R3) complexes in trajectories **1 (left)** and **3 (right)**. OPN-R3 (RNA) is represented as sticks. The RNA loop is in the center **(left)** and on the left-hand side **(right)**. OPN protein is represented as a ribbon with colors associated to secondary motifs identified by the STRIDE (Frishman and Argos, 1995) algorithm (α-helix is in magenta and blue, β-sheet is yellow, turns are in cyano, coils in white); Na ions are yellow spheres, Mg are pink spheres. Atomic and bond radii are arbitrary. The ions were selected as those within a distance of 0.28 nm from any atom of the solute complex. The number of ions (*n*_1_) is 18 for Mg (both configurations) and, respectively, 15 and 16 for Na in trajectory **1 (left)** and **3 (right)**.

The visualization of the configurations of the complex OPN-(OPN-R3) obtained at the end of trajectories **1** and **3** (Figure 5, left and right panels, respectively) shows several common features for the two trajectories.

Both final configurations display the formation of two nascent protein domains. One involves the N-terminal region (first 110 residues), strongly interacting with the RNA aptamer via extended salt bridges involving both Na and Mg cations. Among the 20 available Mg cations, 15 (left panel) and 14 (right panel) ions are concentrated in the assembly of OPN-R3 with the N-terminal OPN domain. The second domain (approximately the C-terminal 174 residues) contains a few cations (3 and 4, respectively, for **1** and **3**). No Cl anion is adsorbed over the assembly. The loop in the stem-loop topology of RNA has different orientations, since in **1** the loop is directed toward the C-terminal domain of OPN (left panel), while the opposite is for **3** (right panel). This shows that the role of RNA sequence is in keeping the stem-loop RNA topology and the density of negative charges of phosphate groups, while the positions of bases have not a strong effect in the assembly topology.

Since the two trajectories are originated from different initial random OPN-(OPN-R3) contacts (Figure S1, Supplementary Material), the formation of the two domains in OPN appears as a common trend for the assembly: the N-terminus of OPN, rich in D and E residues, tends to strongly interact with cations adsorbed by the RNA aptamer; the C-terminus, more hydrophobic, is left for an autonomous protein folding, with a few cations involved. Despite the specificity of this type of assembly, similar long-range folding events are observed in simulations of DNA-protein complexes (Lambrughi et al., 2016). The structures displayed in Figure 5 are atomistic descriptions of the schematic drawings in Figure 1A.

This two-domain behavior of OPN, when the latter is exposed to its RNA aptamer OPN-R3, can be summarized as a folding-upon-binding mechanism, opposite to that proposed for the interaction between OPN and heparin in the absence of divalent cations. Nevertheless, the type of interaction between the OPN N-terminus and RNA is consistent with the few structural results reported in the literature for isolated OPN. The most recent NMR data for non-phosphorylated OPN (Platzer et al., 2015) are consistent with small deviations from random coil values, but the N-terminus displays a long conformationally extended stretch. The propensity for such structural extension in the N-terminus clearly assists the formation of the network of salt bridges displayed in Figure 5.

## 4. Conclusions

The interactions between OPN and its RNA aptamer OPN-R3 are investigated by means of atomistic models where Na and Mg cations are included. The formation of diffuse layers of monovalent cations and more persistent layers of divalent cations around the phosphate groups of RNA, as it is reported in the literature, is confirmed. The negatively charged N-terminus of OPN takes part to this interaction, with cations performing a strong screening of the large negative charge of the nascent assembly.

Several limitations affect the model: (i) the rough model of divalent cations; (ii) the short length of MD trajectories due to the slow conformational change of the disordered protein; (iii) the slow process of exchange of divalent cations. Despite these limitations, the two longer simulations, started from different initial configurations, describe the assembly as two nascent domains: (i) the ternary complex between OPN-R3, the N-terminus of OPN rich in negatively charged residues (D and E), and most of the divalent cations recruited in the solution; (ii) the C-terminus of OPN adopting a collapsed structure where cations are not involved and that points oppositely to the first domain.

This two-domain topology can be altered by OPN phosphorylation, the latter providing a negative charge to other residues (most commonly S, T, and Y) that are more distributed among OPN regions (Lenton et al., 2017). However, this study confirms the importance of electrostatic interactions, showing the further importance of divalent cations in the change of OPN shape, in the location of the aptamer interface, and in the determination of a possible independent domain left open to other types of interactions. The newborn domains in the OPN-(OPN-R3) assembly may provide different functions to OPN, thus providing a general frame for the OPN plasticity.

## Author contributions

All authors listed have made a substantial, direct and intellectual contribution to the work, and approved it for publication.

### Conflict of interest statement

The authors declare that the research was conducted in the absence of any commercial or financial relationships that could be construed as a potential conflict of interest.

## References

[B1] Aaqvist J. (1990). Ion-water interaction potentials derived from free energy perturbation simulations. J. Phys. Chem. 94, 8021–8024. 10.1021/j100384a009

[B2] Casalino L. Palermo G. Abdurakhmonova N. Rothlisberger U. Magistrato A. (2017). Development of site-specific Mg^2+^-rna force field parameters: A dream or reality? Guidelines from combined molecular dynamics and quantum mechanics simulations. J. Chem. Theory and Comput. 13, 340–352. 10.1021/acs.jctc.6b00905 28001405

[B3] Christensen B. Schack L. Kläning E. Sørensen E. S. (2010). Osteopontin is cleaved at multiple sites close to its integrin-binding motifs in milk and is a novel substrate for plasmin and cathepsin d. J. Biol. Chem. 285, 7929–7937. 10.1074/jbc.M109.075010 20071328 PMC2832943

[B4] Cisneros G. A. Karttunen M. Ren P. Sagui C. (2014). Classical electrostatics for biomolecular simulations. Chem. Rev. 114, 779–814. 10.1021/cr300461d 23981057 PMC3947274

[B5] Du J. Hou S. Zhong C. Lai Z. Yang H. Dai J.. (2008). Molecular basis of recognition of human osteopontin by 23c3, a potential therapeutic antibody for treatment of rheumatoid arthritis. J. Mol. Biol. 382, 835–842. 10.1016/j.jmb.2008.07.075 18694758 PMC2793339

[B6] Duarte F. Bauer P. Barrozo A. Amrein B. A. Purg M. Åqvist J.. (2014). Force field independent metal parameters using a nonbonded dummy model. J. Phys. Chem. B 118, 4351–4362. 10.1021/jp501737x 24670003 PMC4180081

[B7] Fennell C. J. Gezelter J. D. (2006). Is the ewald summation still necessary? Pairwise alternatives to the accepted standard for long-range electrostatics. J. Chem. Phys. 124:234104. 10.1063/1.2206581 16821904

[B8] Frangogiannis N. G. (2012). Matricellular proteins in cardiac adaptation and disease. Physiol. Rev. 92, 635–688. 10.1152/physrev.00008.2011 22535894 PMC4411042

[B9] Frishman D. Argos P. (1995). Knowledge-based secondary structure assignment. Proteins 23, 566–579. 10.1002/prot.340230412 8749853

[B10] Hu D. D. Lin E. C. K. Kovach N. L. Hoyer J. R. Smith J. W. (1995). A biochemical characterization of the binding of osteopontin to integrins α_ν_β_1_ and α_ν_β_5_. J. Biol. Chem. 270, 26232–26238. 10.1074/jbc.270.44.26232 7592829

[B11] Humphrey W. Dalke A. Schulten K. (1996). Vmd visual molecular dynamics. J. Mol. Graphics 14, 33–38. 10.1016/0263-7855(96)00018-5 8744570

[B12] Hunter G. K. O'Young J. Grohe B. Karttunen M. Goldberg H. A. (2010). The flexible polyelectrolyte hypothesis of protein-biomineral interaction. Langmuir 26, 18639–18646. 10.1021/la100401r 20527831

[B13] Kahles F. Findeisen H. M. Bruemmer D. (2014). Osteopontin: a novel regulator at the cross roads of inflammation, obesity and diabetes. Mol. Metabolism 3, 384–393. 10.1016/j.molmet.2014.03.004 24944898 PMC4060362

[B14] Kalmar L. Homola D. Varga G. Tompa P. (2012). Structural disorder in proteins brings order to crystal growth in biomineralization. Bone 51, 528–534. 10.1016/j.bone.2012.05.009 22634174

[B15] Kurzbach D. Canet E. Flamm A. G. Jhajharia A. Weber E. M. M. Konrat R.. (2017). Investigation of intrinsically disordered proteins through exchange with hyperpolarized water. Angew. Chem. Int. Ed. 56, 389–392. 10.1002/anie.201608903 27918140

[B16] Kurzbach D. Platzer G. Schwarz T. C. Henen M. A. Konrat R. Hinderberger D. (2013). Cooperative unfolding of compact conformations of the intrinsically disordered protein osteopontin. Biochemistry 52, 5167–5175. 10.1021/bi400502c 23848319 PMC3737600

[B17] Kurzbach D. Schwarz T. C. Platzer G. Höfler S. Hinderberger D. Konrat R. (2014). Compensatory adaptations of structural dynamics in an intrinsically disordered protein complex. Angew. Chem. Int. Ed. 53, 3840–3843. 10.1002/anie.201308389 24604825

[B18] Lambrughi M. De Gioia L. Gervasio F. L. Lindorff-Larsen K. Nussinov R. Urani C.. (2016). Dna-binding protects p53 from interactions with cofactors involved in transcription-independent functions. Nucleic Acids Res. 44, 9096–9109. 10.1093/nar/gkw770 27604871 PMC5100575

[B19] La Penna G. (2003). A constrained maximum entropy method in polymer statistics. J. Chem. Phys. 119, 8162–8174. 10.1063/1.1609197

[B20] La Penna G. Morante S. Perico A. Rossi G. C. (2004). Designing generalized statistical ensembles for numerical simulations of biopolymers. J. Chem. Phys. 121, 10725–10741. 10.1063/1.1795694 15549958

[B21] Lejeune D. Delsaux N. Charloteaux B. Thomas A. Brasseur R. (2005). Protein-nucleic acid recognition: statistical analysis of atomic interactions and influence of dna structure. Proteins 61, 258–271. 10.1002/prot.20607 16121397

[B22] Lenton S. Grimaldo M. Roosen-Runge F. Schreiber F. Nylander T. Clegg R.. (2017). Effect of phosphorylation on a human-like osteopontin peptide. Biophys. J. 112, 1586–1596. 10.1016/j.bpj.2017.03.005 28445750 PMC5406370

[B23] Li S. Wang L. (2012). Phosphorylated osteopontin peptides inhibit crystallization by resisting the aggregation of calcium phosphate nanoparticles. Cryst. Eng. Commun. 14, 8037–8043. 10.1039/C2CE26140E

[B24] Liu J. Lu Y. (2006). Fast colorimetric sensing of adenosine and cocaine based on a general sensor design involving aptamers and nanoparticles. Angew. Chem. Intl. Ed. 118, 96–100. 10.1002/ange.200502589 16292781

[B25] Mazzali M. Kipari T. Ophascharoensuk V. Wesson J. Johnson R. Hughes J. (2002). Osteopontin-a molecule for all seasons. Q. J. Med. 95, 3–13. 10.1093/qjmed/95.1.3 11834767

[B26] Mi Z. Guo H. Russell M. B. Liu Y. Sullenger B. A. Kuo P. C. (2009). Rna aptamer blockade of osteopontin inhibits growth and metastasis of mda-mb231 breast cancer cells. Mol. Therapy 17, 153–161. 10.1038/mt.2008.235 18985031 PMC2834992

[B27] Momma K. Izumi F. (2011). Vesta 3 for three-dimensional visualization of crystal, volumetric and morphology data. J. Appl. Crystallogr. 44, 1272–1276. 10.1107/S0021889811038970

[B28] Oyane A. Kim H.-M. Furuya T. Kokubo T. Miyazaki T. Nakamura T. (2003). Preparation and assessment of revised simulated body fluids. J. Biomed. Mater. Res. 65A, 188–195. 10.1002/jbm.a.10482 12734811

[B29] Peysselon F. Xue B. Uversky V. N. Ricard-Blum S. (2011). Intrinsic disorder of the extracellular matrix. Mol. Biosyst. 7, 3353–3365. 10.1039/C1MB05316G 22009114

[B30] Platzer G. Żerko S. Saxena S. Koźmiński W. Konrat R. (2015). 1H, 15N, 13C resonance assignment of human osteopontin. Biomol. NMR Assignments 9, 289–292. 10.1007/s12104-014-9594-7 PMC4568010 25616494

[B31] Plimpton S. (1995). Fast parallel algorithms for short-range molecular dynamics. J. Comput. Phys. 117, 1–19. 10.1006/jcph.1995.1039.

[B32] Ponta H. Sherman L. Herrlich P. A. (2003). Cd44: from adhesion molecules to signalling regulators. Nat. Rev. Mol. Cell. Biol. 4, 33–45. 10.1038/nrm1004 12511867

[B33] Sodek J. Ganss B. McKee M. D. (2000). Osteopontin. Crit. Rev. Oral Biol. Med. 11, 279–303. 10.1177/10454411000110030101 11021631

[B34] Want J. Cieplak P. Kollman P. A. (2000). How well does a restrained electrostatic potential (resp) model perform in calculating conformational energies of organic and biological molecules? J. Comput. Chem. 21, 1049–1074. 10.1002/1096-987X(200009)21:12<1049::AID-JCC3>3.0.CO;2-F

[B35] Weber G. F. Zawaideh S. Hikita S. Kumar V. A. Cantor H. Ashkar S. (2002). Phosphorylation-dependent interaction of osteopontin with its receptors regulates macrophage migration and activation. J. Leukocyte Biol. 72, 752–761. 10.1189/jlb.72.4.752 12377945

