# Supplementary material for: Structural Insights into the Osteopontin-Aptamer Complex by Molecular Dynamics Simulations
Source: Front Chem. 2018 Jan 30;6:2. doi: 10.3389/fchem.2018.00002 (PMC5797602; doi:10.3389/fchem.2018.00002)
Supplement: Supplementary file 1 [file DataSheet1.pdf]

Supplementary Material: Structural insights into the osteopontin-aptamer complex by molecular dynamics simulations

1 SUPPLEMENTARY DATA

In this document, we provide:

- the details about the construction of the initial OPN-R3 structure and OPN-(OPN-R3) complex structures;
- the details about the simulations performed;
- a summary of structural parameters used in analysis;
- the details of the results about the OPN-R3 aptamer.

2 CONSTRUCTION OF INITIAL STRUCTURES

2.1 Construction of the initial OPN-R3 configuration

First the OPN-R3 aptamer was simulated in the absence of OPN. The initial configuration for the OPN-R3 aptamer was built with the MFold algorithm Zuker (2003). The algorithm produces the expected stem-loop configuration, ruled by the formation of the largest number of canonical U-A and C-G base-pairs. The RNA molecule was placed into a cubic cell with the side-length of 15 nm, filled of water molecules with the density according to the molecular dynamics (MD) simulation of the TIP3P model Jorgensen et al. (1983). Water molecules with O atom within 0.12 nm from any RNA atom were discarded. Water molecules were randomly replaced by Na and Cl ions up to obtaining an approximate 0.1 M concentration, while keeping the charge neutralization of the simulation cell. The “solvate” and “autoionize” tools included in the version 1.9.3 of VMD program Humphrey et al. (1996) were used for this set-up. The system size is summarized in Table 1 of the main document. To obtain the sample with a concentration of Mg one order of magnitude lower than Na, the set-up was repeated adding the same number of Na ions, 20 Mg ions (each ion composed by 7 point-charges, see below), and a lower number of Cl ions to keep the charge neutrality.

The energy of the initial configurations was minimized. The AMBER PARM99 force field Want et al. (2000) was used, because of the amount of results reported for RNA structures by using this force field Leontis and Westhof (2012). This force field was used for both protein and RNA in all the simulations described in this work. As for cations, we used a recent force field for divalent cations Duarte et al. (2014), in addition to the usual parametrization of NaCl Aaqvist (1990). This set-up reduces the charge density over the divalent cation, thus avoiding the usual problem of negatively charged (carboxylate and phosphate) groups collapsing over the highly-charged points.

All the simulations follow the same equilibration protocol described below and were performed using the LAMMPS code Plimpton (1995). The distance cut-off of 1.1 nm was used for both Lennard-Jones and Coulomb interactions. The Coulomb interactions were screened with a damping parameter α =2 nm^{-1} (*i.e.*, charges were screened according to a Gaussian smoothing with a width of 2 nm^{-1}). We used the damped and shifted force method Fennell and Gezelter (2006) implemented in LAMMPS. For a critical

review about this method compared to more accurate methods see Ref. 5. We used the velocity-Verlet algorithm to integrate the equation of motions, first with a time-step of 0.5 fs (for 10 ps), then with a time-step of 2 fs (for 20 ps) by constraining bond lengths involving H atoms with the velocity-compliant SHAKE algorithm Andersen (1983). Keeping the volume constant, a Nosé-Hoover thermostat Shinoda et al. (2004) was added for 20 ps at 150 K and for 400 ps at 300 K, with a temperature damping parameter of 100 fs. Finally, an isotropic barostat was added with a target pressure of zero and a damping parameter of 1 ps. The simulation of the complex at the constant temperature T =300 K and zero pressure was then performed for about 20 ns for each of the two systems, respectively with Mg and without Mg.

As for the construction of initial configurations for the OPN-(OPN-R3) complex, the configuration of RNA with Mg at 18 ns was extracted. This configuration, as shown by the analysis described below, has 9 Mg cations adsorbed. These cations were kept for the construction of the initial complex samples.

2.2 Construction of the initial OPN configuration

We built a random configuration for the OPN protein. The OPN sequence used in our model is that reported for the OPN-b precursor (expressed by SPP1 gene in *Homo sapiens*), with the first 16 residues removed, as in the first post-translational modification Sodek et al. (2000). Therefore, our sequence starts with I(1)PV and ends with EVN(284). All the P residues are kept in the *trans* conformation and all the aminoacid side-chains are protonated according to physiological pH of 7.4: D and E are deprotonated and negatively charged; K and R are protonated and positively charged; H residues are protonated in Nϵ2 and neutral (Nϵ2-H tautomer). No further post-translational modifications are taken into account.

The OPN initial configuration was built with all Φ and Ψ dihedral angles, except Φ of P, set to 180°. Then an empirical force field was chosen to compute interatomic energies and forces, in order to sample, via Monte Carlo (MC) and MD methods, configurations of OPN-RNA complexes compatible with the room conditions used for *in vitro* experiments. Once force field and initial configuration are chosen for each model, a set of non overlapping configurations was generated by using random temperature MC trajectories, summarized in the following.

In MC trajectories, the sampling of configurations with gyration radius closer to the value for peptides in water solution was enhanced by using a 0.5 nm cut–off for non bonding interactions. As in the simulation of OPN-R3 in water solution, the electrostatic interactions were screened according to a Gaussian charge smoothing with a width of 2 nm^{-1}, and were shifted for neutralization Wolf et al. (1999). This algorithm greatly reduces energy fluctuations due to pair–potential truncation errors. In the charge neutralization, only non bonding atoms are taken into account.

In a Metropolis MC simulation of a single molecular chain Frenkel and Smit (1996), the configuration is changed by modifications of dihedral angles not involved in rings (like phenyl and imidazole aromatic groups). A randomly chosen dihedral angle in the chain is set to a dihedral angle randomly chosen within zero and 360° (we limited the selection of dihedral angles to those not involving H atoms). The temperature for the Metropolis MC test, based on the potential energy change upon the configuration modification, is randomly chosen within 0 and a given maximum, 10,000 K in the present case. The inverse temperature $\beta = 1/(RT)$ was uniformly sampled in the corresponding temperature range. The trajectory of accepted configurations describes a metastatistics, that is the statistics resembling the density of states in the given force–field La Penna (2003); La Penna et al. (2004). These trajectories are indicated as MC random-walks (MC-RW hereafter). Overlapping configurations are avoided by the Metropolis test used in the MC-RW chain. Since with this set-up the acceptance ratio, r, is in the 0.3-0.5 range, for each MC-RW the sampling frequency for configurational analysis was always N_{deg}/r, where N_{deg} is the number of modified dihedral

angles in the MC-RW. As for OPN, the configuration obtained after 10,000 MC-RW iterations was used as initial configuration of building tentative first-encounter OPN-(OPN-R3) complex structures.

2.3 Construction of the initial OPN-(OPN-R3) complex

The initial configuration for OPN-R3, including 9 Mg cations adsorbed on its surface, was oriented with the stem axis approximately aligned with the z-axis of the laboratory reference frame, with the stem close to the origin and the loop pointing towards z. The chosen initial configuration for OPN was then placed in several points close to the center of the OPN-R3 loop. We placed the C-terminus of OPN (C atom of N284) at an approximate distance of 3 nm from the loop center in RNA (P of A19). Then we moved the C atom of N284 in other 5 positions, translating of 5 nm along x and y axis.

After this initial construction, the dihedral Φ and Ψ angles of the OPN protein were randomly modified starting from the C-terminus. The MC-RW was repeated starting from each of the 6 initial configurations manually built. Thus, the C-terminus (N284) and the RNA aptamer were kept fixed in space in each of the 6 samples, while the entire protein chain of OPN randomly moves around the RNA molecule. We choose the first configuration displaying a manifest contact between OPN and OPN-R3 as the starting configuration for the following MD simulation. Depending on the initial configuration, 500-1000 MC-RW iterations were sufficient to achieve such initial wrapping of OPN around the chosen RNA aptamer configuration including the 9 Mg cations. The configurations of the 6 selected samples are displayed in Fig. S1. The other ions and water molecules were added using the same procedure adopted for OPN-R3 (see above).

Therefore, once a configuration mimicking a possible initial contact between OPN and RNA was selected within the 6 MC-RW performed, MD simulations of the OPN-(OPN-R3) complex were performed in explicit water and with explicit counterions, following the procedure adopted for the RNA aptamer OPN-R3 (see above). In order to mimic the solution environment in the extracellular compartment where OPN is more active, we modeled three regimes of concentrations for divalent cations. The concentration of Na^{+} is always within 114 and 127 mM, while the concentration of Mg^{2+} varies from 6 mM to zero in three steps. The non-zero Mg concentrations are consistent with the range of concentrations for divalent cations (Mg^{2+} and Ca^{2+}) in the simulated body fluid Oyane et al. (2003) used for *in vitro* OPN-RNA binding Mi et al. (2009) and biomineralization studies Kalmar et al. (2012). To slightly increase the rate of OPN-RNA association in simulations, in the first stage (about 10 ns) of simulation the number of Mg cations adsorbed on the RNA surface was kept to $\sim$9, as found for isolated RNA fragment simulations with the same simulation set-up. In the second part of the simulation (further 10 ns), the Mg^{2+} cations that are found forming salt-bridges between OPN and RNA were replaced with Na^{+} ions, with insertion in bulk water of the number of Na ions required to neutralize the simulation box. A final stage of about 10 ns is performed with no Mg cations. After performing 10 ns of simulation in the NPT statistical ensemble for the 6 samples, each at the 3 different salt concentrations, we performed longer simulations (55 ns) for two selected samples and conditions.

2.4 Analysis

The number of contacts is defined as a continuous variable with the following equation Barducci et al. (2006):

$$\begin{aligned} CN &= \sum_{i,j} s_{i,j} \ ; \\ s_{i,j} &= 1 \text{ if } r_{i,j} \leq 0 \\ &= 0 \text{ if } r_{i,j} > 0 \ ; \\ r_{i,j} &= |\mathbf{r}_i - \mathbf{r}_j| - d_0 \ . \end{aligned} \tag{S1}$$

with i and j running over the heavy atoms contained in different portions of the system (RNA, OPN, Mg or Na ions, etc.). The d_0 parameter is defined according to the peaks identified in the radial distribution functions for the respective sets of atoms. For each pair, the distance d_0 was chosen as the distance containing the first peak of the relative pair distribution function. The number of contacts is related to the charge (Q_c) cumulated in the contact layers of OPN and OPN-R3, provided each ion identified by i is counted only once when the contact is multidentate. The index j runs over the selected atoms of OPN and RNA. In several configurations one ion can be involved in a salt bridge with one RNA ligand atom on one side and one OPN ligand atom on the other side. In these cases, the contact would be counted twice and the charge neutralization effect would be overestimated. To avoid this error, we define the quantity $n_1(\mathrm{M})$ as the number of cations M contained in the first shell identified by the radial distribution function $g(r)$ for X$-$M distance, with X running over atoms belonging to different solute portions.

Hydrogen bonds are defined when the X$-$Y distance in X$-$H$\cdots$Y is smaller than 0.3 nm and the X$-$H$\cdots$Y angle is larger than 135°.

The solvent-accessible surface area (SASA) and solvent-excluded volume (molecular volume, hereafter) were measured with the algorithm described in Ref. 7. Atomic radii for RNA atoms were hinerited by equivalent groups in aminoacids Ooi et al. (1987). The radius of P was set to 0.21 nm.

We shall use electron units for charge, hereafter.

The extent of complex formation is measured via the ratio R between the SASA of the complex and the sum of the SASA of the two partners, OPN and OPN-R3, respectively:

$$\mathrm{R} = \mathrm{SASA}([\mathrm{OPN}\text{-}(\mathrm{OPN}\text{-}\mathrm{R3})])/[\mathrm{SASA}(\mathrm{OPN}) + \mathrm{SASA}(\mathrm{OPN}\text{-}\mathrm{R3})] \ . \tag{S2}$$

2.5 RNA aptamer (OPN-R3)

2.5.1 Assessing equilibration

It is now well known that MD simulations require long times, in the range of μs, to sample the conformational landscape of macromolecules Galindo-Murillo et al. (2015). In this work we address the equilibration of the ionic atmosphere around the few biased structures of OPN-R3 and (OPN-R3)-OPN complex.

Scalar quantities that well represent the change of shape of a macromolecule in a homogeneous solvent environment are the solvent-accessible surface area (SASA, A) and solvent-excluded volume (V, see Methods). We measured these quantities for OPN-R3 in NaCl and NaCl/$MgCl_2$ solutions along the NPT-ensemble simulations (see Fig. S2, left panel, for SASA, V displays an identical behavior and it is not

shown here). The time-evolution of SASA shows that after a rapid decrease occurring in about 1 ns A starts a slow oscillation. The rapid decrease is associated with the rapid decrease of the simulation cell volume, that also occurs during the first ns of the NPT-ensemble MD simulation (data not shown here). The slow 10-ns range oscillation can be associated with the change of structure in the disordered loops of OPN-R3, in particular the region 34-40. This type of structural disorder is displayed by the range for P(i)-P(j) distances involving nucleotides that are facing in the reference ideal double strand. These distances are 2.1÷0.6, 2.1÷0.4 and 2.7÷4.5 nm for $i - j$ pairs 3-33, 14-21 and 2-40, respectively, thus showing that the P−P distance reflect a molecular wobbling in those regions forming base-pairs, while closed and open conformations are sampled for dangling single strands (34-40, see hydrogen bond analysis below).

The SASA in the presence of Mg cations is different than in NaCl (right curve in Fig. S2, left panel). The first assessment of SASA takes at least 10 ns, compared to 5 ns in the presence of monovalent cations. Then, the oscillation is around a lower value, showing a more compact RNA average structure when in the presence of divalent cations, an effect that has been observed in experiments Moghaddam et al. (2009). The contribution to the reduction of SASA is mainly due to the 3'-terminus (nucleotides 34-40) that with Mg becomes closed towards the ordered stem (see Fig. S4 and discussion below).

The analysis of the time evolution of root-mean square deviation (RMSD) Kabsch (1976) of configuration at time t with respect to the initial, mostly canonical, configuration (Fig. S2, right panel), displays that without Mg cations the RMSD oscillates between 0.4 and 0.7 nm after 5 ns, when the segment 34-40 is not included (red curve). The addition of segment 34-40 increases the oscillation range to 0.5-0.8 nm, but still the RMSD displays a steady-state behavior. When Mg cations are added to the solution (Fig. S2, right panel, right curves), the structure is more rigid at the beginning, but displays a slow drift towards the higher values displayed when no Mg is in solution. This drifts of RMSD affects also the 1-33 segment (red curve), thus it is not caused by the 34-40 dangling segment (see above). This shows that upon the slow adsorption of Mg by RNA (see discussion below of Fig. S7), the RNA stem structure is slowly affected by a strong electrostatic screening.

This structural analysis for OPN-R3 in NaCl solution allows to discard the first 5 ns from the following analysis. Using the last 16.2 ns of the OPN-R3 trajectory in NaCl, we can approximate the RNA molecule as a cylinder with volume V =20.3±0.2 nm^3 and radius R (estimated by wobbling P(i)-P(j) distance above) of 2.1/2=1.05 nm. An approximate heigth h can be obtained as $h = V/(\pi R^2) =$ 5.9 nm. When Mg cations are present, since the RNA molecule has on average a smaller SASA, but the same average P-P distances, the same calculation provides h =5.8 nm. Therefore, we shall assume 6 nm as an average height for the RNA approximating cylinder in both cases.

2.5.2 Base-pairing

The analysis of hydrogen bonds within the bases of OPN-R3 allows to describe the deviation of the RNA structure from the initial model, the latter constructed on the basis of canonical B-DNA base-pairs (see Methods). Fig. S3 displays graphically the inter-base hydrogen bonds measured over the last 16.2 ns for OPN-R3 in NaCl (left panel) and over 18.4 ns for OPN-R3 with Mg (right panel), mapped onto the RNA sequence. The lines connect nucleotides involved in inter-base hydrogen bonds. Segments are displayed when the hydrogen bond population is larger than 50%. The figure displays that in NaCl the base-pairing of OPN-R3 is kept from 3-33 to 14-21, while this stem structure is distorted in the presence of Mg. This base-pairing disorder introduced by Mg is related to the extent of Mg adsorption. In the final configuration obtained with Mg, we observe a significant number of Mg ions adsorbed by RNA phosphate groups, while only a few Na ions are adsorbed in the same region (see Fig. S4). This results in the distortions of the

second stem segment (bases 5'-(6-8)-3' paired with 5'-(28-30)-3'), causing a small sliding of base 6. When Mg is absent, only a few Na ions are loosely adsorbed on RNA (see below) and the backbone distortions are released.

2.5.3 Ionic layers

The hydrogen bonds analysis reported above confirms the characterization of OPN-R3 shape that allows to simplify the OPN-R3 molecule as a cylinder. The geometrical parameters estimated above can be safely used both with and without Mg. Therefore, it is possible to approximate the OPN-R3 fragment as a cylinder with a radius R =1.1 nm, height h =6.0 nm and a net charge $Q = -39$.

Even if structurally more disordered than B-DNA, the charge density of RNA is in the same range of that of B-DNA and, therefore, a significant extent of counterion condensation over the OPN-R3 fragment is expected. This possibility is also emphasized by the presence in the simulated body fluid, used for all binding measurements, of divalent cations. The possibility for RNA to attract cations is exploited by the MD simulation of the RNA fragment in the absence of OPN protein and it is described below.

The analysis of counterion density around the RNA fragment is performed according to the work reported in Ref. 15. Each of the configurations of the RNA fragment is inscribed into the cylinder described above, with the center located in the center of mass of the RNA atoms. The long axis is defined as the eigenvector of the RNA inertia tensor with minimal eigenvalue. The distance between ions and the center of the cylinder was measured and the probability for a distance was measured as a function of the cylinder radius r. The probability to have the distance within a given distance range, $N_{pe}(r)$, was then divided for the same probability for the ideal gas with the same uniform ion density, $N_{pe,id}(r)$, thus defining the radial distribution function $g_{pe}(r) = N_{pe}(r)/N_{pe,id}(r)$. The height h of the cylinder was chosen as 6.5 nm, according to the structural analysis reported above. With this construction, it is possible to estimate the radius of the cylinder where the concentration of ions approach the bulk value, *i.e.* when $r = R_{bulk}$.

In Fig. S5 the above-defined $g_{pe}(r)$ function is displayed for Na ions, averaging over two time-windows (0-8.1 ns and 8.1-16.2 ns, the thinner lines) of the trajectory and collecting the data of the 16.2 ns trajectory (the thick line). The structure of the function within 3 nm is different in the two samples, because the ions are slowly settling the interactions with RNA, but the absence of structure beyond 3 nm is already converged in the first half of the trajectory. This length is defined as R_{bulk}, the approximate size of the condensation layer. We measured the amount of charge Q due to Na and Cl within the cylinder of height 6.5 nm and radius $r = R_{bulk}$, averaged over the last 16.2 ns. The effective charge $Q_{eff} = Q(\mathrm{OPN\text{-}R3}) + Q(\mathrm{Na}) + Q(\mathrm{Cl}) = -39 + 30 - 6 = -15$ in the absence of Mg. This result means that most of the net charge of RNA is screened beyond a distance of 3 nm from the center of the cylinder approximating the RNA molecule.

Condensation theory Manning (1978) predicts the screening of 3/4 of the net charge of an infinitely long charged line with the same charge density of B-DNA and at physiological ionic conditions (0.1 M in NaCl). Therefore the screened charge according to Manning's theory is $Q_{eff} = -9.8$ when projected to the small fragment of RNA of 40 bases. Since the RNA fragment is different from the straight and infinitely long B-DNA, the agreement between the two results, in the absence of Mg, is fairly good.

When Mg ions are added to the water solution, the competition between divalent and monovalent ions changes the structure of the condensation layer. In Fig. S6, we show the $g_{pe}(r)$ function for Na (red), Mg (green) and Cl (blue) in the simulation of OPN-R3 in the presence of Mg. The comparison between Na and Mg shows that Mg displays a strong adsorption over phosphate groups, that are at

about 1 nm from the long RNA axis. The Na ions are definitely more sparsed within the approximate condensation layer (r <3 nm). The distribution of Na is smoother than that observed in the absence of Mg (see Fig. S5), showing that the displacement of Na by Mg from the first-shell of phosphate groups, keeps the Na ions in a farther and looser shell of RNA. Mg cations are not adsorbed deeper in the RNA grooves, since distance smaller than 0.5 nm is rarely sampled. Due to this difference, the integral of the $N_{pe}(r)$ function from zero to 3 nm approximating the number of RNA-condensed ions (see above) gives a change of the effective charge Q_{eff} (see above for the case with no Mg). The effective charge $Q_{eff} = Q(\mathrm{RNA} + Q(\mathrm{Na}) + Q(\mathrm{Cl}) + Q(\mathrm{Mg}) = -39 + 21 - 6 + 18 = -6$ in the presence of Mg. In this case, most of the screening is due to the adsorption of 9 Mg cations, compared to 21 Na ions (30 in the absence of Mg). These data show that Mg replaces Na in the condensation layer, but the Mg ions are more structured (a narrower peak of $g_{pe}(r)$) around the phosphate groups of RNA.

It is worth comparing Fig. S6 with Fig.2 of Ref. 15. In the latter work the same function is displayed for monovalent (Na and Rb) and divalent (Mg and Sr) cations around an infinite RNA double strand in the A-form. These simulations display a more pronounced structure compared to those reported in this work for both Na and Mg cations within a similar condensation distance (3 nm). In particular both Na and Mg cations penetrate more deeply in the RNA chain and divalent cations display weaker interactions with phosphate groups (peaks at $r \sim$1 nm in Fig.2 are lower than in this work, Fig. S6). However, we notice that in our model the concentration of Mg is lower than in the previous simulations (10 mM compared to 200 mM). As for differences in Na distribution, the smoother g_{pe} function observed for OPN-R3 both in the absence (Fig. S5) and presence (Fig. S6) of Mg is due to the more disordered stem-loop structure of OPN-R3 compared to A-RNA. The bending of the stem-loop topology (see Fig. S4) shifts some of the close-contact peaks from distances of $\sim$1 nm to distances of $\sim$2 nm away from the axis of the cylinder approximating the RNA solute.

In order to better describe the difference in structure between Na and Mg ions around the OPN-R3 fragment, we measured the number of short-range contacts between cations and the RNA solute. Therefore, we counted the number of cations $n_1(\mathrm{M})$ (see Methods) in the first shell identified by the radial distribution function $g(r)$ for X$-$M distance, with X either any atom in the RNA solute or O in the phosphate oxygen atom not involved in the phospho-diester linkage of RNA (OP, hereafter). M is, respectively, Na and Mg. This shell has the size d_0 (Eq. S2) that depends on the pair of atoms involved. By analyzing the $g(r)$ functions for X$-$M pairs, the parameter d_0 is chosen as 0.28 nm for all pairs.

In Fig. S7, the number of OP-adsorbed M (n_1), with M=Na and Mg, is displayed as a function of the simulation time. The simulation with no Mg is displayed as the continuation of that with Mg. The RNA molecule adsorbs 8$\pm$2 divalent cations and 2$\pm$1 Na cations, on average. The comparison of $g(r)$ for OP-M pairs with that for all pairs X$-$M shows that this number is dominated by OP contacts when M is Mg, but there is a significant difference when M=Na. Even when Mg is removed, the number of X-adsorbed Na is 6$\pm$2, while that for OP-adsorbed Na is 3$\pm$2. This difference means that Na ions are not really adsorbed, while there is exchange between the first shell and farther shells, allowing short-range interactions with groups different from the phosphate groups. This observation is consistent with a loose structure of short-range Na shells. On the other hand, in case of Mg, the number of adsorbed ions measures the extent of ion adsorption by the phosphate groups as an additional information to the ion condensation, the latter occurring within the larger R_{bulk} distance (3 nm, see above).

A tendency of exchanging Na with Mg is observed in the time-range of 10 ns: the number of adsorbed Na ions decreases after 9 ns in the simulation with Mg, as a consequence of a slow penetration of Mg into the condensation layer. In the absence of Mg the number of phosphate-adsorbed Na cations increases to

an average of 6±2, restoring the loose Na cloud that was present before the replacement of Na by Mg occurring in the simulation with Mg.

Most of the screening contribution due to Mg is made by phosphate-adsorbed Mg (8 cations over 9, see above). This adsorption is a very slow process, as already noticed in 100-ns long simulations Casalino et al. (2017). Na is more diffuse within the distance R_{bulk} and the phosphate-adsorbed Na ions are only a small and sparsed contribution to the screening positive charge (2 over 22 Na ions within R_{bulk}). The structure of these layers will serve as a reference for the comparison with the OPN-aptamer complex described in the following.

3 SUPPLEMENTARY FIGURES

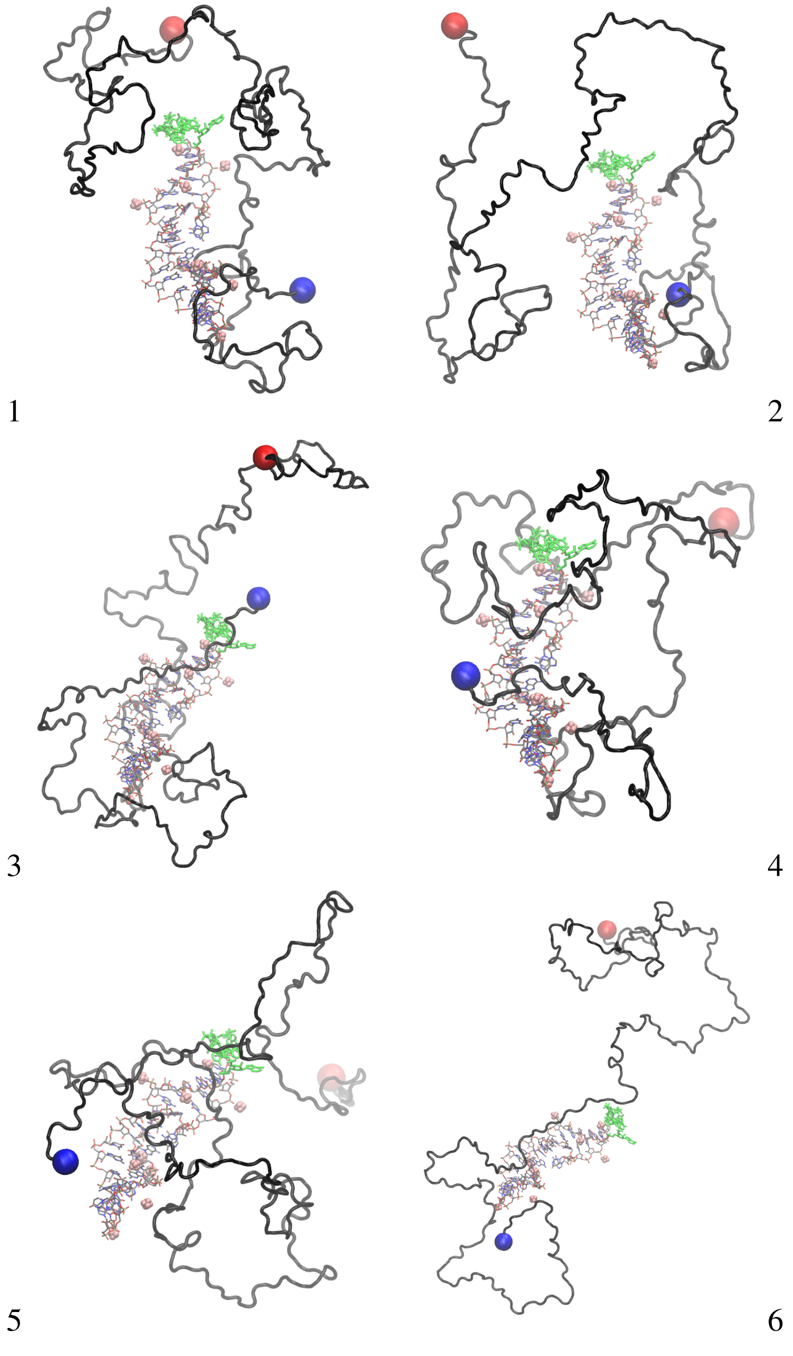

Figure S1: Initial configurations of OPN-(OPN-R3) for MD simulations of 6 samples. RNA is represented as sticks; C is grey, N blue, O red; H atoms are not displayed; nucleotides 15-20 are in green, to emphasize the RNA loop; the 9 Mg ions kept from RNA simulation are displayed as pink octahedrons; the OPN protein is represented as a black tube; the C- and N-termini in OPN are displayed as red and blue spheres, respectively. Bond, atomic and tube radii are arbitrary. The OPN-R3 and Mg configurations are all identical, the orientation is chosen to better display the initial configurations. The VMD program Humphrey et al. (1996) is used for all the molecular drawings.

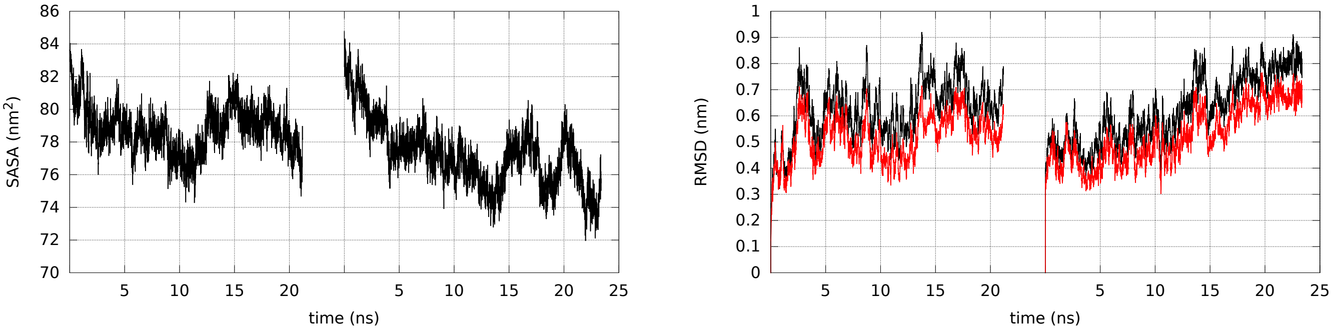

Figure S2: Time-evolution of SASA (left panel) of OPN-R3 simulated with NaCl 121 mM (left curve) and with NaCl 121 mM and $MgCl_2$ 10 mM (right curve). Time-evolution of root-mean square deviation (RMSD) calculated for all RNA atoms (black curve) and for nucleotides 1-33 (red curve).

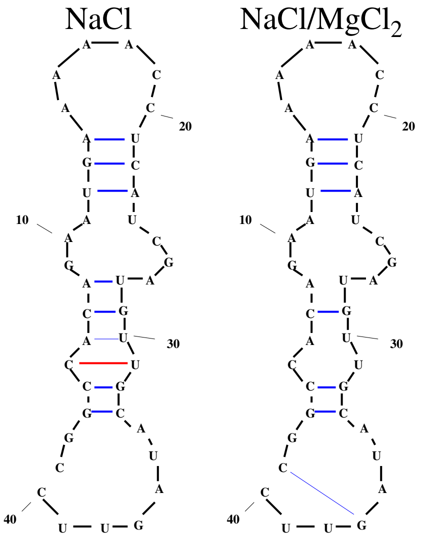

Figure S3: Base-pairs hydrogen bonds in OPN-R3 aptamer displayed as those with population $P \geq 50\%$. Blue thick lines represent hydrogen-bonds consistent with the canonical AU/CG base-pairing (initial RNA structure), thin lines represent lower number of hydrogen bonds, red lines represent non-canonical base pairing. RNA with no Mg (left) and with Mg (right) (see text for details).

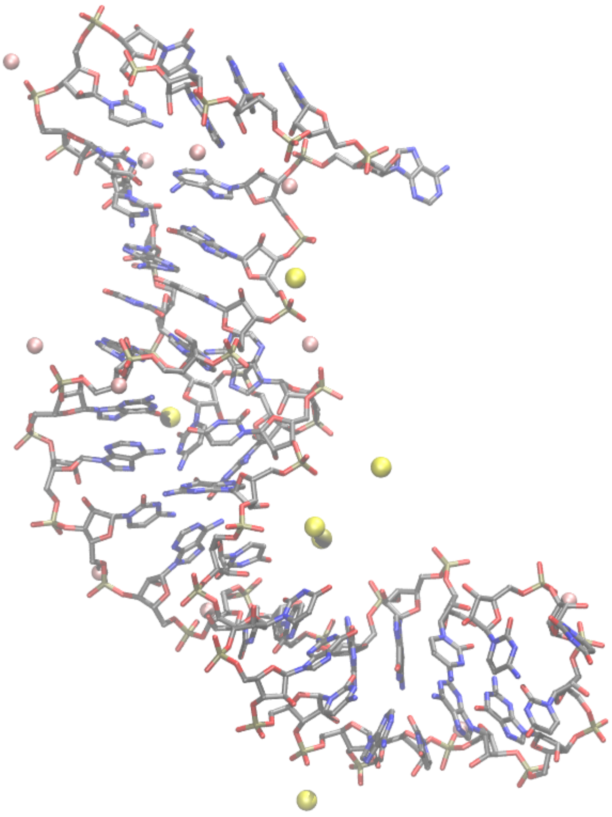

Figure S4: Final configuration of OPN-R3 simulated for 23.4 ns with $MgCl_2$. RNA is represented as sticks; C is grey, N blue, O red; H atoms are not displayed; Mg and Na ions within 0.5 nm from OP are represented as pink and yellow spheres, respectively. Bond and atomic radii are arbitrary. The stem-loop RNA region (11-24) is on top of the figure and the RNA termini (1-2 and 34-40) are on the bottom-right.

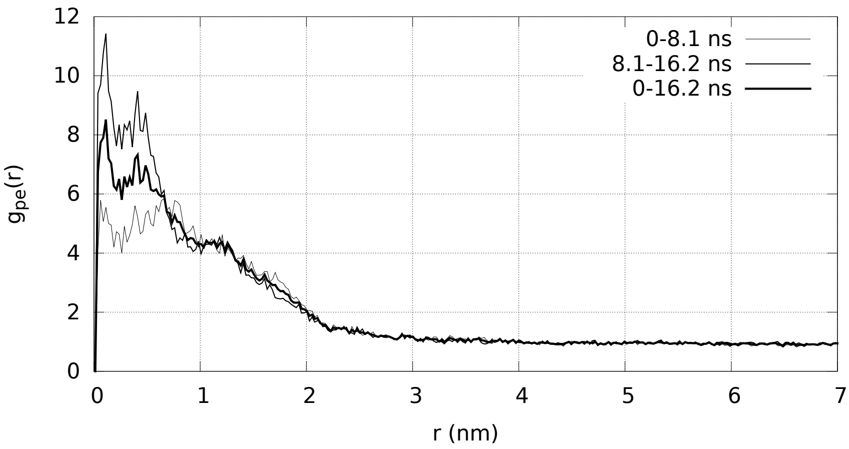

Figure S5: Distribution (g_{pe}) of distance r between Na ions and the axis of the cylinder representing the OPN-R3 molecule (see text for details) in the absence of Mg. Thin lines are distribution evaluated for the two halves of the simulation, thick line is for the whole 16.2 ns trajectory after 5 ns of equilibration.

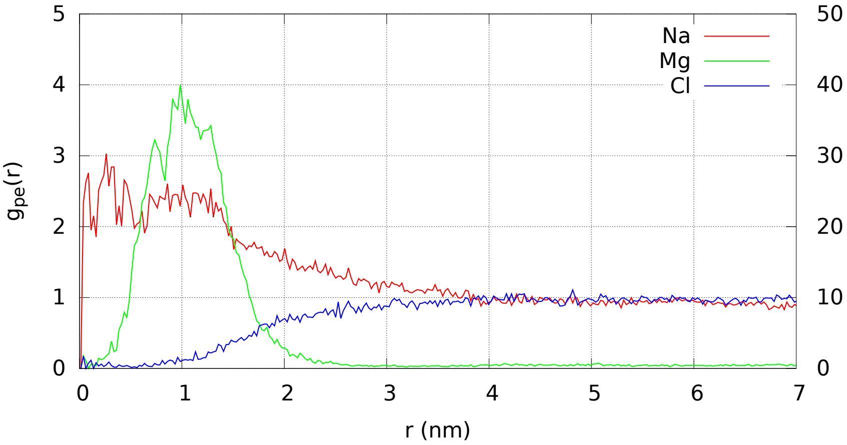

Figure S6: Distribution (g_{pe}) of distance r between Na (red), Mg (green) and Cl (blue) ions and the axis of the cylinder representing the OPN-R3 molecule (see text for details) in the presence of Mg for the whole 13.4 ns trajectory after 10 ns of equilibration. The right y-axis refers to the curve for Mg.

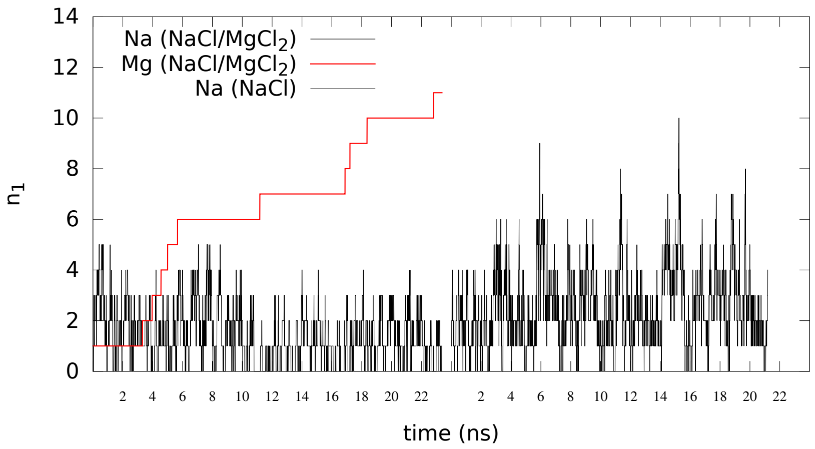

Figure S7: Time evolution of OP-adsorbed M ions (d_0 =0.28 nm) with M=Na (thin line) and Mg (thick line). The evolution in the absence of Mg is displayed as continuation of that with Mg.

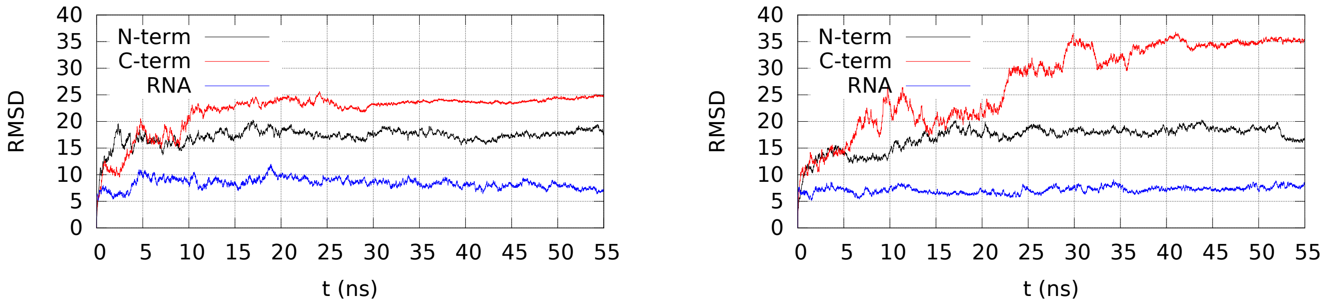

Figure S8: Time evolution, within 55 ns of simulation, of root-mean square deviation (RMSD) for OPN atoms (black curve residues 1-173, red curve residues 174-284) with respect to initial structure, and RNA atoms (blue curve) with respect to initial canonical structure. Left panel is for trajectory **1** and right panel for trajectory **3**.

REFERENCES

Aaqvist, J. (1990). Ion-water interaction potentials derived from free energy perturbation simulations. *J. Phys. Chem.* 94, 8021–8024. doi:10.1021/j100384a009

Andersen, H. C. (1983). Rattle: A velocity version of the shake algorithm for molecular dynamics. *J. Comput. Phys.* 52, 24–34. doi:10.1.1.459.5668

Barducci, A., Chelli, R., Procacci, P., Schettino, V., Gervasio, F. L., and Parrinello, M. (2006). Metadynamics simulation of prion protein: β-structure stability and the early stages of misfolding. *J. Am. Chem. Soc.* 128, 2705–2710. doi:10.1021/ja057076l

Casalino, L., Palermo, G., Abdurakhmonova, N., Rothlisberger, U., and Magistrato, A. (2017). Development of site-specific mg^{2+}-rna force field parameters: A dream or reality? guidelines from combined molecular dynamics and quantum mechanics simulations. *J. Chem. Theory and Comput.* 13, 340–352. doi:10.1021/acs.jctc.6b00905

Cisneros, G. A., Karttunen, M., Ren, P., and Sagui, C. (2014). Classical electrostatics for biomolecular simulations. *Chem. Rev.* 114, 779–814. doi:10.1021/cr300461d

Duarte, F., Bauer, P., Barrozo, A., Amrein, B. A., Purg, M., Åqvist, J., et al. (2014). Force field independent metal parameters using a nonbonded dummy model. *J. Phys. Chem. B* 118, 4351–4362. doi:10.1021/jp501737x

Eisenhaber, F., Lijnzaad, P., Argos, P., Sander, C., and Scharf, M. (1995). The double cubic lattice method: Efficient approaches to numerical integration of surface area and volume and to dot surface contouring of molecular assemblies. *J. Comput. Chem.* 16, 273–284. doi:10.1002/jcc.540160303

Fennell, C. J. and Gezelter, J. D. (2006). Is the ewald summation still necessary? pairwise alternatives to the accepted standard for long-range electrostatics. *J. Chem. Phys.* 124, 234104. doi:10.1063/1.2206581

Frenkel, D. and Smit, B. (1996). *Understanding Molecular Simulation* (San Diego, USA: Academic Press)

Galindo-Murillo, R., Roe, D. R., and Cheatham III, T. E. (2015). Convergence and reproducibility in molecular dynamics simulations of the dna duplex d(gcacgaacgaacgaacgc). *Biochim. Biophys. Acta* 1850, 1041–1058. doi:10.1016/j.bbagen.2014.09.007

Humphrey, W., Dalke, A., and Schulten, K. (1996). Vmd visual molecular dynamics. *J. Molec. Graphics* 14, 33–38. doi:10.1016/0263-7855(96)00018-5. Http://www.ks.uiuc.edu/Research/vmd

Jorgensen, W. L., Chandrasekhar, J., Madura, J. D., Impey, R. W., and Klein, M. J. (1983). Comparison of simple potential functions for simulating liquid water. *J. Chem. Phys.* 79, 926–935. doi:10.1063/1.445869

Kabsch, W. (1976). A solution for the best rotation to relate two sets of vectors. *Acta Cryst.* A32, 922–923. doi:10.1107/S0567739476001873

Kalmar, L., Homola, D., Varga, G., and Tompa, P. (2012). Structural disorder in proteins brings order to crystal growth in biomineralization. *Bone* 51, 528 – 534. doi:10.1016/j.bone.2012.05.009

Kirmizialtin, S., Pabit, S., Meisburger, S., Pollack, L., and Elber, R. (2012). Rna and its ionic cloud: Solution scattering experiments and atomically detailed simulations. *Biophys. J.* 102, 819–828. doi:10.1016/j.bpj.2012.01.013

La Penna, G. (2003). A constrained maximum entropy method in polymer statistics. *J. Chem. Phys.* 119, 8162–8174. doi:10.1063/1.1609197

La Penna, G., Morante, S., Perico, A., and Rossi, G. C. (2004). Designing generalized statistical ensembles for numerical simulations of biopolymers. *J. Chem. Phys.* 121, 10725–10741. doi:10.1063/1.1795694

Leontis, N. and Westhof, E. (eds.) (2012). *RNA 3D Structure analysis and prediction*. Nucleic Acids and Molecular Biology (Berlin Heidelberg, D: Springer-Verlag). doi:10.1007/978-3-642-25740-7

Manning, G. S. (1978). The molecular theory of polyelectrolyte solutions with applications to the electrostatic properties of polynucleotides. *Q. Rev. Biophys.* 11, 179–246

Mi, Z., Guo, H., Russell, M. B., Liu, Y., Sullenger, B. A., and Kuo, P. C. (2009). Rna aptamer blockade of osteopontin inhibits growth and metastasis of mda-mb231 breast cancer cells. *Mol. Therapy* 17, 153–161. doi:10.1038/mt.2008.235

Moghaddam, S., Caliskan, G., Chauhan, S., Hyeon, C., Briber, R., Thirumalai, D., et al. (2009). Metal ion dependence of cooperative collapse transitions in rna. *J. Mol. Biol.* 393, 753–764. doi:10.1016/j.jmb.2009.08.044

Ooi, T., Oobatake, M., Némethy, G., and Scheraga, H. A. (1987). Accessible surface areas as a measure of the thermodynamic parameters of hydration of peptides. *Proc. Natl. Acad. Sci. USA* 84, 3086–3090

Oyane, A., Kim, H.-M., Furuya, T., Kokubo, T., Miyazaki, T., and Nakamura, T. (2003). Preparation and assessment of revised simulated body fluid. *J. Biomed. Mater. Res.* 65A, 188–195. doi:10.1002/jbm.a.10482

Plimpton, S. (1995). Fast parallel algorithms for short-range molecular dynamics. *J. Comput. Phys.* 117, 1–19. doi:10.1006/jcph.1995.1039. Http://www.lammps.sandia.gov

Shinoda, W., Shiga, M., and Mikami, M. (2004). Rapid estimation of elastic constants by molecular dynamics simulation under constant stress. *Phys. Rev. B* 69, 134103. doi:10.1103/PhysRevB.69.134103

Sodek, J., Ganss, B., and McKee, M. D. (2000). Osteopontin. *Crit. Rev. Oral Biol. Med.* 11, 279–303. doi:10.1177/10454411000110030101

Want, J., Cieplak, P., and Kollman, P. A. (2000). How well does a restrained electrostatic potential (resp) model perform in calculating conformational energies of organic and biological molecules? *J. Comput. Chem.* 21, 1049–1074. doi:10.1002/1096-987X(200009)21:12<1049::AID-JCC3>3.0.CO;2-F

Wolf, D., Keblinski, P., Phillpot, S. R., and Eggebrecht, J. (1999). Exact method for the simulation of coulombic systems spherically truncated, pairwise r-1 summation. *J. Chem. Phys.* 110, 8254–8282. doi:10.1063/1.478738

Zuker, M. (2003). Mfold web server for nucleic acid folding and hybridization prediction. *Nucleic Acids Res.* 31, 3406–3415. doi:10.1093/nar/gkg595
